# Ohio State Dental Association

**Published:** 1868-12

**Authors:** 


### Editorial.

### OHIO STATE DENTAL ASSOCIATION.

The annual meeting of this body was held on Tuesday and Wednesday, 1st and 2d of December.

There were about thirty members, with a goodly number of visitors, present. A very warm interest was manifested in the work and objects of the Society.

The discussions, of which there was an unusually large amount, as will be seen by the report, were very interesting and instructive. We think no one who was present during the session failed to receive valuable practical knowledge.

It was a matter of considerable regret, at the time, that there was not more written essays; but, in looking over the whole work after the close, it is difficult to see how the time could have been much better employed. In associative effort like this, the mere communication and reception of knowledge are not the only things to be accomplished. We all need stimulating to a higher and more thorough use of the knowledge we already possess. This is perhaps oftentimes far more difficult of accomplishment than the communication of new truth. We think it is quite obvious to any one, who is disposed to view the matter dispassionately, that this Society is exercising a very salutary influence upon the profession of our State; perhaps not accomplishing all in this respect it might, but it has certainly done a great deal, and we trust it will gather strength and influence from year to year for the accomplishment of still greater good. T.

